# Comparative Transcriptome Analyses Reveal a Special Glucosinolate Metabolism Mechanism in *Brassica alboglabra* Sprouts

**DOI:** 10.3389/fpls.2016.01497

**Published:** 2016-10-04

**Authors:** Rongfang Guo, Zhongkai Huang, Yanping Deng, Xiaodong Chen, Xu XuHan, Zhongxiong Lai

**Affiliations:** ^1^Department of Horticulture, Fujian Agriculture and Forestry UniversityFuzhou, China; ^2^Institute of Horticultural Biotechnology, Fujian Agriculture and Forestry UniversityFuzhou, China; ^3^Institut de la Recherche Interdisciplinaire de ToulouseToulouse, France

**Keywords:** glucosinolate, sprouts, myrosinase, *Brassica alboglabra*, Chinese kale

## Abstract

*Brassica* sprouts contain abundant phytochemicals, especially glucosinolates (GSs). Various methods have been used to enhance GS content in sprouts. However, the molecular basis of GS metabolism in sprouts remains an open question. Here we employed RNA-seq analysis to compare the transcriptomes of high-GS (JL-08) and low-GS (JL-09) *Brassica alboglabra* sprouts. Paired-end Illumina RNA-seq reads were generated and mapped to the *Brassica oleracea* reference genome. The differentially expressed genes were analyzed between JL-08 and JL-09. Among these, 1477 genes were up-regulated and 1239 down-regulated in JL-09 compared with JL-08. Enrichment analysis of these differentially expressed genes showed that the GS biosynthesis had the smallest enrichment factor and the highest *Q*-value of all metabolic pathways in Kyoto Encyclopedia of Genes and Genomes database, indicating the main metabolic difference between JL-08 and JL-09 is the GS biosynthetic pathway. Thirty-seven genes of the sequenced data were annotated as putatively involved in GS biosynthesis, degradation, and regulation, of which 11 were differentially expressed in JL-08 and JL-09. The expression level of GS degradation enzyme myrosinase in high-GS JL-08 was lower compared with low-GS JL-09. Surprisingly, in high-GS JL-08, the expression levels of GS biosynthesis genes were also lower than those in low-GS JL-09. As the GS contents in sprouts are determined by dynamic equilibrium of seed stored GS mobilization, *de novo* synthesis, degradation, and extra transport, the result of this study leads us to suggest that efforts to increase GS content should focus on either raising GS content in seeds or decreasing myrosinase activity, rather than improving the expression level of GS biosynthesis genes in sprouts.

## Introduction

Chinese kale (*Brassica alboglabra*), a cruciferous biennial vegetable plant originating in southern China, is known for its tender bolting stem and leaves and is mainly produced in Fujian and Guangdong Provinces. Besides good flavor, Chinese kale also has abundant antioxidants including glucosinolates (GSs; Sun et al., 2011a,b, 2012). In recent years, sprouts of Chinese kale have also been consumed and market demand is increasing owing to their unique flavor and higher GS content compared with the mature plant (Wei et al., 2011). Chemo-protective properties of GSs and their hydrolysis products against chemical carcinogens in organs including the liver, bladder, pancreas, colon, and small intestine have been well-demonstrated (Higdon et al., 2007; Razis et al., 2011; Jeffery, 2014). These compounds act as potent chemo-preventive agents by promoting apoptosis in cancer cells or by inhibiting cell cycle progression, some of which have been used in clinical trials, demonstrating their potential in drug development against various cancers (Vig et al., 2009).

GSs are a diverse group of sulfur-rich anionic natural products of plant in the order Capparales. They are grouped into aliphatic, aromatic, and indolic glucosinolates, based on whether they are derived from aliphatic amino acids (often methionine), phenylalanine or tyrosine, or tryptophan, respectively (Halkier and Gershenzon, 2006). GSs are chemically stable unless they come in contact with myrosinases (b-thioglucoside glucohydrolases, EC 3.2.3.1), which are stored in different cellular compartments to separate them from the glucosinolates. Upon tissue damage, glucosinolates are released from the vacuoles and are rapidly hydrolyzed to glucose and thiohydroximate-O-sulfonate by myrosinases. The latter one is unstable and spontaneously become isothiocyanates, thiocyanates, nitriles, epithionitriles, or oxazolidine-2-thiones (Fahey et al., 2001).

GS biosynthetic pathways have been elucidated in *Arabidopsis thaliana* (Grubb and Abel, 2006; Halkier and Gershenzon, 2006). The amino acid tryptophan and methionine are substrates of indolic and aliphatic GS biosynthesis, respectively, in shoots of *A. thaliana*, which were first converted into the corresponding aldoximes by CYP79B2/CYP79B3 and CYP79F1/CYP79F2, respectively (Yan and Chen, 2007). The subsequent conversion of aldoximes to S-alkylthiohydroximates is further catalyzed by CYP83B1 and CYP83A1 (Bak and Feyereisen, 2001). The cleavage of S-alkylthiohydroximates by C-S lyase produced thiohydroximates (Mikkelsen et al., 2004). These are glycosylated by UDP glycosyltransferases to desulfoglucosinolates (Gachon et al., 2005), then sulfated by sulfotransferases (Piotrowski et al., 2004) subsequently. MYB34, MYB51, and MYB122 as well as MYB28, MYB29, and MYB76 are six transcriptional factors defined as regulators of GS biosynthesis in *A. thaliana*. The former three MYBs regulate biosynthesis of indolic GS (Celenza et al., 2005) whereas the latter three MYBs regulate aliphatic biosynthesis of GS (Gigolashvili et al., 2007b, 2008).

Although the main GS biosynthetic pathway is similar in *Brassicaceae*, the regulation of glucosinolate synthesis is very complex in *Brassica* vegetable crops compared with *Arabidopsis*. In *Brassica rapa*, three *BrMYBs* have been found to share 81–87% similarity in coding sequence compared to *Arabidopsis AtMYB28*. It was reported that *BrAOP2* was negatively regulated and *BrGS-OH* was positively regulated in *BrMYB28.1*-overexpressed lines by all three *BrMYB28s*, indicating a different regulatory mechanism of GS biosynthesis in *B. rapa* compared with *A. thaliana* (Seo et al., 2016). In *B. juncea, CYP79F1* shows presence–absence polymorphism between lines. Genetic and transgenic approaches have been used to validate that the biosynthesis of 3-carbon (3C) GS can be regulated by *CYP79F1* in *B. juncea* (Sharma et al., 2016). However, *GSL-PRO* is a probable candidate gene responsible for 3C GS biosynthesis in *A. thaliana, Brassica napus*, and *B. oleracea* (Magrath et al., 1994; Li et al., 2003).

Substantial quantities of GSs have been found in *Brassica* sprouts which are 7–9 days after germination (Fahey et al., 1997; Cartea and Velasco, 2008). Various methods for enhancing sprout quality by increasing their GS content have been tried, including treatment with different qualities and quantities of light (Kopsell and Sams, 2013; Vale et al., 2015b), induction by sucrose and glucose (Guo et al., 2011; Wei et al., 2011), different processing (Ciska et al., 2015), varying sprouting phase length (Vale et al., 2015a), and post-harvest techniques (Vale et al., 2015b). However, little is known about the mechanism of GS accumulation in *Brassica* sprouts.

Transcriptome methods are based on next-generation sequencing of RNA (RNA-seq) which can acquire the gene sequences and identify transcripts involved in specific biological processes. RNA-seq has been used widely in identifying genes involved in kinds of secondary metabolites biosynthetic pathways (Wang et al., 2013), including carotenoid biosynthetic pathways in *Momordica cochinchinensis* (Hyun et al., 2012), cellulose and lignin biosynthesis in Chinese fir (Huang et al., 2012), tea-specific flavonoid, theanine and caffeine biosynthetic pathways in tea (Shi et al., 2011), biosynthesis of flavonoids in safflower (Lulin et al., 2012), and biosynthesis of capsaicinoids in chilli pepper (Liu et al., 2013). We selected two *B. alboglabra* varieties in our lab with significantly different GS content in their sprouts and used these to identify the genes involved in GS biosynthetic and degradation pathway. In the present study, RNA-seq is performed on *B. alboglabra* was performed for the first time using Illumina sequencing. Combined with quantitative analysis and bioinformatics prediction, we figure out genes related to GS metabolic pathway in *B. alboglabra* and propose a possible mechanism of GS in its sprouts.

## Materials and methods

### Plant material

Fifty-five varieties of Chinese kale (*B. alboglabra*) were collected from South China and then self-crossed for five generations. After identification of glucosinolates profiles content in sprouts, two lines JL-08 and JL-09 were noticed for their different glucosinolates content and used for the following analysis. Seeds of JL-08 and JL-09 were disinfected in sodium hypochlorite (0.7%) for 30 min then drained and washed seven times with distilled water. After soaked for 24 h, seeds with broken seed coat were selected to grow in 15 cm petri dishes laid with three layers of wet filter paper. The filter paper was wet by 15 mL distilled water before sowing and added by 10 mL distilled water every 3 days after sowing. Four petri dishes with 100 seeds each for JL-08 and JL-09, respectively were planted and put in an incubator (25°C) under a 16/8 h (light/dark) photoperiod. Finally, 7-day-old sprouts were sampled from different petri dishes and stored at −80°C for GS analysis and RNA extraction. At least three biological replications were used in the following measurement, respectively.

### Measurement of GS content

GSs were extracted and analyzed as previously described with minor modifications (Guo et al., 2011). Samples (500 mg) were boiled in 3 mL water for 10 min. After transferring the supernatant to a new tube, the residues were washed with water (3 mL), and the combined aqueous extract was applied to a DEAE-Sephadex A-25 (30 mg) column (pyridine acetate form; Sigma, St. Louis, MO, USA). The column was washed three times with 20 mM pyridine acetate and twice with water. The glucosinolates were converted into their desulfo analogs by overnight treatment with 100 μL of 0.1% (1.4 units) aryl sulphatase (Sigma, St. Louis, MO, USA) added into the column, and the desulfoglucosinolates were collected by eluting with 2 × 0.5 mL water. HPLC analysis was performed using an HPLC system consisting of an Agilent HPLC series chromatograph (Agilent Technologies). The same C18 column and procedure was used as described in Guo et al. (2011). The peak was detected at 226 nm. Ortho-nitrophenyl-β-d-galactopyranoside (Sigma, St. Louis, MO, USA) was used as an internal standard. The glucosinolate content was calculated as μmol/g fresh weight.

### RNA extraction, library construction, and RNA-seq

RNA of Chinese kale sprouts was extracted from JL-08 and JL-09 six times for three biological repeats, respectively, using Trizol Reagent (Invitrogen). After characterization of RNA purity by Nanodrop 1000 spectrophotometer (Thermo Fisher Scientific, Wilmington, DE, USA) and measurement of RNA concentration by Qubit® 2.0 Flurometer (Life Technologies, CA, USA), RNA integrity was assessed by Agilent Bioanalyzer 2100 system (Agilent Technologies, CA, USA). RNA samples with integrity number more than 7.0 were selected to construct libraries. Illumina sequencing was performed at Biomarker Technologies Corporation (Beijing, China) following procedures similar to Lou et al. (2014). After enrichment and purification with oligo(dT)-rich magnetic beads, mRNA was interrupted into short fragments, which are converted to the first- and second- strand cDNA. The cDNA was purified by AMPure XP beads, and repaired at 3′ ends of cDNA fragments, then added poly (A) and ligated to adapters for selection of a size range of templates. Finally, the six cDNA libraries were enriched by PCR amplification and sequenced using an Illumina HiSeq™ 2500.

### RNA-seq reads mapping and transcript assembly

After removing those with only adaptor and unknown nucleotides larger than 5%, or those that were of low quality, the clean reads were filtered from the raw reads. Cleaned RNA-seq reads were then mapped to the reference genome http://plants.ensembl.org/Brassica_oleracea/Info/Index using Bowtie (http://bowtie-bio.sourceforge.net/index.shtml) and TopHat2 (http://ccb.jhu.edu/software/tophat/index.shtml; Kim et al., 2013). Then the SAM (Sequence Alignment Map; http://samtools.sourceforge.net/; Li et al., 2009) files were generated by TopHat2 and subsequently transcripts were assembled by Cufflinks (http://cufflinks.cbcb.umd.edu/; Trapnell et al., 2010). Fragments per Kilobase of exon per Million Fragments (FPKM) was used to measure Cuffdiff to describe transcript abundance.

### Expression annotation

Gene function was annotated based on the following databases: Nr (NCBI non-redundant protein sequences), Pfam (Protein family), COG (Clusters of Orthologous Groups of proteins), Swissprot (A manually annotated and reviewed protein sequence database), KEGG (Kyoto Encyclopedia of Genes and Genomes database), and GO (Gene Ontology). The SOAP aligner (http://soap.genomics.org.cn/soapaligner.html) was used to evaluate the coverage depth of reads. Differential expression analysis of two varieties was performed using the ratio of the FPKM values. Reads abundance with a *P* < 0.05 were assigned as differentially expressed based on False Discovery Rate (FDR) control. The unique reads with the value of logarithms of radio ≥ 1 and FDR < 0.01 were assigned as differentially expressed genes (DEGs). GO and KEGG enrichment analysis of the DEGs were implemented later.

### Gene validation and expression analysis

The qPCR was performed to validate results got from RNA-seq of glucosinolate biosynthesis related genes. RNA samples were reverse-transcribed into cDNA using PrimeScript® RT reagent Kit (Takara Code: DRR037A). Expression profiles of genes were examined in triplicate using SYBR® Premix Ex Taq™ II (Tli RNaseH Plus) (Takara Code: RR820A) in LightCycler 480 (Roche Applied Science, Switzerland) following 25 μL Real Time system including 12.5 μL SYBR® Premix Ex Taq II (2X), 1.0 μL Forward and Reverse Primer (10 μM) with the final concentration of 0.4 μM, respectively, 2.0 μL cDNA (50 ng/μL), and 8.5 μL sterile distilled water. Two-step PCR was performed according to the manufacturer's procedure and the initial denaturation step is 95°C for 30 s, followed by 40 cycles of 95°C for 5 s and 60°C for 30 s. The *Acting* was used as the internal control and the primers used were listed in Supplementary Table 1.

### Statistical analysis

Statistical analysis was performed using the SPSS package program version 19.0 (SPSS, Chicago, IL, USA). The data were analyzed by one-way analysis of variance. The values are reported as means ± standard error (SE) for all results. Differences were considered significant at *P* < 0.05.

## Results

### Profiles and contents of GSin chinese kale sprouts

**Figure 1:**
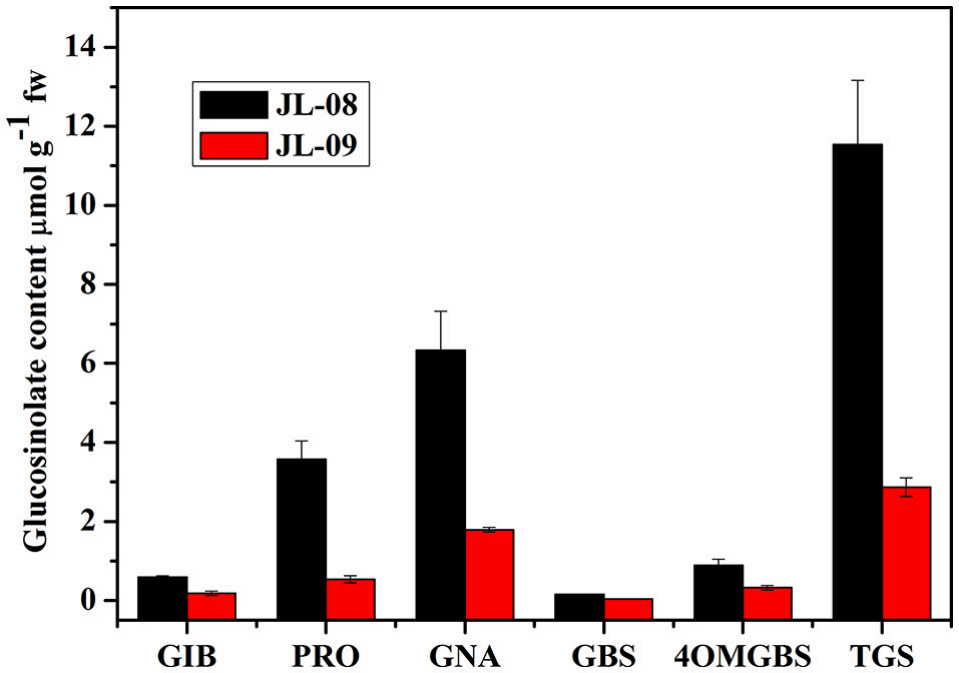
**Profiles and contents of GS in JL-08 and JL-09 sprouts**. The *X* axis represented the profiles of glucosinolate in JL-08 and JL-09 sprouts. GIB, Glucoiberin; PRO, Progoitrin; GNA, Gluconapin; GBS, Glucobrassicin; 4OMGBS, 4-Methoxy glucobrassicin; TGS, Total glucosinolate. The *Y* axis is the content of individual and total glucosinolate in JL-08 and JL-09 sprouts. Black column, JL-08; Red column, JL-09. The error bar represents the ± SE.

To examine the biochemical basis of GS diversity in *B. alboglabra*, we compared the profiles and content of sprouts of two varieties. As expected, JL-08 and JL-09 sprouts mainly contain three kinds of aliphatic glucosinolates—glucoiberin (GIB), progoitrin (PRO), and gluconapin (GNA) as well as two kinds of indolic glucosinolates—glucobrassicin (GBS) and 4-methoxyglucobrassicin (4-OMGBS; Figure 1). The aliphatic GSs predominate with proportions of 91.1% in JL-08 and 87.1% in JL-09. GNA is the main aliphatic glucosinolate in Chinese kale, accounting for 60.4% in JL-08, and 71.4% in JL-09. The high-performance liquid chromatogram of JL-09 showed greatly reduced levels of total glucosinolate as well as of individual ones compared with JL-08, and thus we refer to JL-09 as low-GS Chinese kale and to JL-08 as high-GS Chinese kale.

### RNA-seq analysis of chinese kale sprouts aligned with the *Brassica oleracea* reference genome sequence

**Figure 2:**
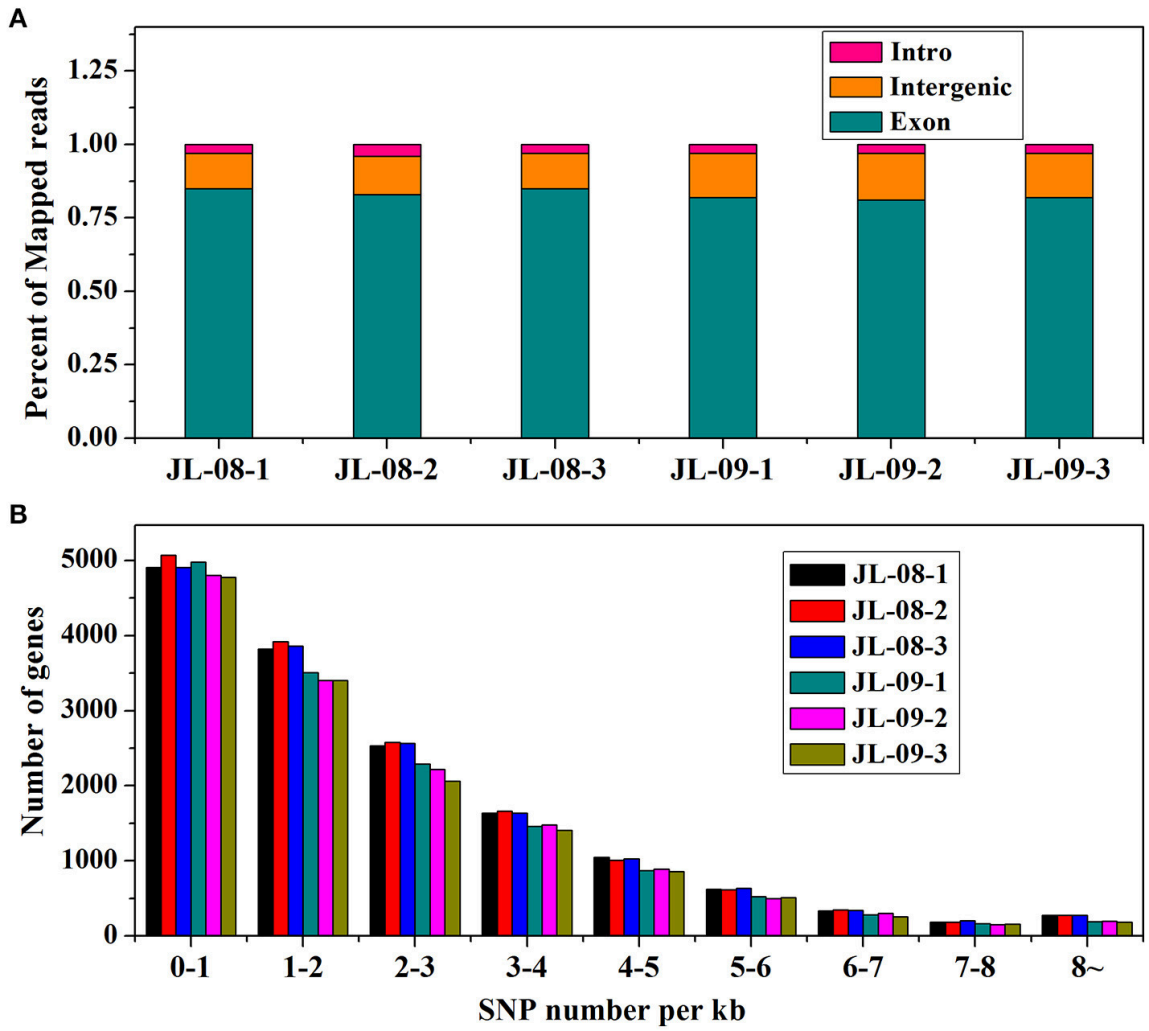
**(A)** The percent of mapped reads distributed in different sites (intro, intergenic, and exon) of gene in JL-08 and JL-09. The *X* axis represented three repetitions of sequenced JL-08 and JL-09, respectively. JL-08-1, JL-08-2, and JL-08-3 are three repetitions of JL-08. JL-09-1, JL-09-2, and JL-09-3 are three repetitions of JL-09. The *Y* axis is the percent of mapped reads distributed in different sites of gene. Pink column: intro; Yellow: intergenic; Cyan: extron. **(B)** The SNP density values on specific length gene in JL-08 and JL-09 sprouts. The X axis represented nine gene length ranges (0–1 kb, 1–2 kb, 2–3 kb, 3–4 kb, 4–5 kb, 5–6 kb, 6–7 kb, 7–8 kb, and 8~ kb). The left axis is the number of SNP loci based on gene length in three repetitions of sequenced JL-08 and JL-09, respectively. JL-08-1, JL-08-2, and JL-08-3 are three repetitions of JL-08. JL-09-1, JL-09-2, and JL-09-3 are three repetitions of JL-09. Black, JL-08-1; Red, JL-08-2; Blue, JL-08-3; Cyan, JL-09-1; Pink, JL-09-2; Yellowish green, JL-09-3.

RNA sequencing of six samples (three replicates each of JL-08 and JL-09 sprouts) was performed on the Illumina HiSeq2500 sequencing platform. After removal of adaptor sequences, ambiguous reads, and low-quality reads, ~148 million 251-bp paired-end reads were produced (an average of 25 million reads for each sample), comprising 37,302,596,852 nucleotides (37.3 Gb). As shown in Table 1, the Q20 percentages (sequencing error rates lower than 1%) are from 93.62 to 93.88% and GC percentages are from 47.64 to 47.98%. After aligned with *B. oleracea* reference genome GCA_000695525.1 (http://plants.ensembl.org/Brassica_oleracea/Info/Index) and subsequent analysis using TopHat2, 45,088,551 (80.96%), 46,341,039 (80.11%), and 48,637,840 (80.82%), in three JL-08 replicates as well as 33,805,444 (82.98%), 33,277,163 (83.17%), and 34,684,524 (83.21%) reads in three JL-09 replicates were mapped to the *B. oleracea* reference genome, respectively. Among these, 78.58, 77.42, and 78.40% in three JL-08 replicates as well as 80.86, 80.77, and 80.86% in three JL-09 replicates were mapped uniquely to one location. More than 85% of mapped reads were in exons (Figure 2A).

**Table 1:** **Statistics of reads generated by transcriptome sequencing of *B. alboglabra* sprouts**.

**Sample ID**	**ReadSum**	**BaseSum**	**GC (%)**	**Q20 (%)**	**Q30 (%)**	**Total reads**	**Mapped reads**	**Mapped ratio (%)**	**Uniq mapped reads**	**Uniq mapped ratio (%)**
JL-08-1	27844528	7014683023	47.92	93.73	88.87	55689056	45088551	80.96	42757808	78.58
JL-08-2	28924525	7285494707	47.75	93.62	88.72	57849050	46341039	80.11	44787229	77.42
JL-08-3	30090034	7579911708	47.9	93.88	89.14	60180068	48637840	80.82	47178889	78.40
JL-09-1	20370684	5132236431	47.75	93.7	88.46	40741368	33805444	82.98	32943876	80.86
JL-09-2	20004512	5039842842	47.64	93.63	88.73	40009024	33277163	83.17	32314637	80.77
JL-09-3	20840388	5250428141	47.98	93.62	88.54	41680776	34684524	83.21	33702856	80.86

To elucidate gene structures, single nucleotide polymorphisms (SNPs) and alternative splicing were predicted. SNPs were detected using SAM tool, the numbers of SNPs compromising in genic and intergenic regions are listed in Table 2. According to the base substitutions observed, SNP loci can be classified as transitions or transversions. The percentage of each type was calculated, and the transversion rate is higher than the transition rate in all samples. SNP density values were calculated as the number of SNP loci divided by the length of the gene (Figure 2B).

**Table 2:** **Gene structures in terms of single nucleotide polymorphism (SNP) and alternative splicing**.

**BMK-ID**	**SNP number**	**Genic SNP**	**Intergenic SNP**	**Transition (%)**	**Transvertion (%)**	**Skipped exon**	**Intron retention**
JL-08-1	85,042	65,177	19,865	58.37	41.63	2164	5879
JL-08-2	87,093	66,052	21,041	58.33	41.67	2237	6293
JL-08-3	86,308	65,571	20,737	58.28	41.72	2272	6079
JL-09-1	73,173	58,190	14,983	58.34	41.66	1867	5423
JL-09-2	72,464	57,329	15,135	58.37	41.63	1845	5811
JL-09-3	69,181	55,383	13,798	58.43	41.57	1574	4258

Alternative splicing can be divided into six types: exon skipping, intron retention, alternative 5′ splice site, alternative 3′ splice site, alternative last exon, and alternative first exon. The numbers of predicted alternative splicing events of each type are listed in Table 3. Intron retention is the most common type of alternative splicing predicted one in all samples, with up to 6293 instances.

**Table 3:** **Biosynthetic Genes related to glucosinolate biosynthesis in *B. alboglabra***.

**GS pathway**	**Gene name**	**ID**	**JL-08 Ave ± Se**	**JL-09 Ave ± Se**	**KEGG_annotation**	**Nr_annotation**	**Regulated**
Chain elongation	BCAT4	Bo3g073430	0.00 ± 0.00	2.35 ± 0.62	K00826|1e-156|ath:AT3G19710|BCAT4	Branched-chain aminotransferase 4 [*B. rapa* subsp. *pekinensis*]	Up
		Bo5g113720	0.00 ± 0.00	28.00 ± 3.01	K00826|0.0|ath:AT3G19710|BCAT4	Branched-chain aminotransferase 4 [*B. rapa* subsp. *pekinensis*]	Up
	BAT5	—	—	—	—	—	—
	MAM1	Bo2g161100	0.00 ± 0.00	17.85 ± 0.72	K01649|0.0|ath:AT5G23010|MAM1	2-isopropylmalate synthase [*B. oleracea*]	Up
		Bo7g098000	0.00 ± 0.00	14.50 ± 2.04	K01649|0.0|ath:AT5G23010|MAM1	2-isopropylmalate synthase, B genome specific 2 [*B. juncea*]	Up
	IPMI	—	—	—	—	—	—
	IPMDH	—	—	—	—	—	—
	BCAT3	Bo1g080200	25.23 ± 1.62	19.94 ± 0.55	K00826|0.0|ath:AT3G49680|BCAT3	ATBCAT-3 [*A. lyrata* subsp. lyrata]	No
		Bo8g078930	18.17 ± 0.24	18.55 ± 0.66	K00826|0.0|ath:AT3G49680|BCAT3	Branched-chain aminotransferase 3 [*B. rapa* subsp. *pekinensis*]	No
Core structure formation	CYP79F1	Bo5g021810	0.00 ± 0.00	26.30 ± 3.04	K12154|0.0|ath:AT1G16410|CYP79F1	CYP79F1 [*B. oleracea*]	Up
	CYP79F2	—	—	—	—	—	—
	CYP79B2	Bo3g152800	3.59 ± 1.59	11.61 ± 2.33	K11812|0.0|ath:AT4G39950|CYP79B2	Cytochrome P450 79B1 [*B. oleracea* var. *botrytis*]	No
		Bo1g002970	27.76 ± 2.50	33.99 ± 4.87	K11812|0.0|ath:AT4G39950|CYP79B2	Cytochrome P450 [*B. napus*]	No
		Bo7g118840	11.42 ± 1.86	14.03 ± 2.04	K11812|0.0|ath:AT4G39950|CYP79B2	Cytochrome P450 79B1 [*B. oleracea* var. *botrytis*]	No
	CYP79B3	Bo4g149550	1.93 ± 1.02	1.30 ± 0.16	K11813|0.0|ath:AT2G22330|CYP79B3	Cytochrome P450 79b3 [*B. rapa* subsp. *pekinensis*]	Up
	CYP79A2	Bo9g177260	0.07 ± 0.01	0.00 ± 0.00	K12153|0.0|ath:AT5G05260|CYP79A2	Cytochrome P450 79a2 [*B. rapa* subsp. *pekinensis*]	No
	CYP83A1	Bo4g130780	7.08 ± 1.28	186.7 ± 7.00	K12156|0.0|ath:AT4G13770|CYP83A1	Cytochrome P450 monooxygenase 83A1-5 [*B. napus*]	Up
		Bo4g191120	0.10 ± 0.03	2.87 ± 0.23	K12156|0.0|ath:AT4G13770|CYP83A1	Cytochrome P450 83a1 [*B. rapa* subsp. *pekinensis*]	No
	CYP83B1	Bo8g024390	173.49 ± 8.04	194.63 ± 16.84	K11818|0.0|ath:AT4G31500|CYP83B1	Cytochrome P450 monooxygenase 83-5 [*B. napus*]	No
	SUR1	Bo7g003330	30.37 ± 5.13	39.64 ± 1.87	K11819|0.0|ath:AT2G20610|SUR1	SUR1 [*B. rapa* subsp. *pekinensis*]	No
	UGT74B1	Bo5g041080	15.23 ± 1.58	30.69 ± 1.98	K11820|0.0|ath:AT1G24100|UGT74B1	Glucosyltransferase [*B. oleracea*]	No
	UGT74C1	—	—	—	—	—	—
	SOT	Bo6g118380	88.62 ± 1.29	65.52 ± 11.36	K11821|0.0|ath:AT1G74100|SOT16	Sulfotransferase 5a [*B. rapa* subsp. *pekinensis*]	No
Side chain modification	AOP2	—	—	—	—	—	—
	AOP3	—	—	—	—	—	—
degradation	TGG	Bo8g039420	2.67 ± 0.67	63.33 ± 2.19	—	Myrosinase, thioglucoside glucohydrolase [*B. napus*]	Up
		Bo00934s010	0.00 ± 0.00	1.67 ± 0.33	—	Myrosinase [*B. oleracea* var. *alboglabra*]	No
		Bo2g155810	1267.00 ± 77.31	2560.00 ± 63.00	—	Myrosinase [*B. oleracea* var. *alboglabra*]	No
		Bo9g022660	18.00 ± 1.53	30.00 ± 6.51	K01238|3e-30|ath:AT5G24550|BGLU32	Myrosinase [*B. napus*]	No
		Bo14804s010	65.67 ± 3.84	47.00 ± 5.86	—	Myrosinase [*B. napus*]	No

New genes were predicted using Cufflinks by stitching the mapped reads and comparing to the original genome annotation. After filtering out short peptides (less than 50 amino acid residues) and single exon sequences, a total of 1633 new genes were predicted.

### Differentially expressed gene analysis in chinese kale sprouts with different GS content

Gene expression levels were normalized to the number of FPKM. Because the difference in GS content between varieties might be caused by differential genes expression, we performed an analysis of DEGs and found that 2716 genes of which 1477 up-regulated and 1239 down-regulated (Table 4).

**Table 4:** **Transcriptional factors related to glucosinolate biosynthesis in *B. alboglabra***.

**Gene name**	**ID**	**KEGG_annotation**	**Swissprot_annotation**	**Nr_annotation**	**Regulated**
MYB28	Bo2g161590	K09422|0.0|ath:AT5G61420|MYB28	Transcription factor MYB28 GN = MFB13.22 OS = *Arabidopsis thaliana* (Mouse-ear cress) PE = 1 SV = 1	Myb transcription factor *BoMyb28-1* [*Brassica oleracea* var. *viridis*]	Up
	Bo9g014610	K09422|0.0|ath:AT5G61420|MYB28	Transcription factor MYB28 GN = MFB13.22 OS = *Arabidopsis thaliana* (Mouse-ear cress) PE = 1 SV = 1	Myb transcription factor *BoMyb28-2* [*Brassica oleracea* var. *viridis*]	Up
	Bo7g098590	K09422|0.0|ath:AT5G61420|MYB28	Transcription factor MYB28 GN = MFB13.22 OS = *Arabidopsis thaliana* (Mouse-ear cress) PE = 1 SV = 1	Myb domain protein 28 [*Brassica oleracea* var. *italica*]	Up
MYB29	Bo3g004500	K09422|1e-161|ath:AT5G07690|ATMYB29 (ARABIDOPSIS THALIANA MYB DOMAIN PROTEIN 29)	Transcription factor MYB29 GN = MBK20.15 OS = *Arabidopsis thaliana* (Mouse-ear cress) PE = 1 SV = 1	MYB domain protein 29-1 [*Brassica rapa* subsp. *pekinensis*]	No
MYB76	Bo6g118710	K09422|1e-129|ath:AT1G74430|MYB95	Transcription factor MYB76 GN = MBK20.16 OS = *Arabidopsis thaliana* (Mouse-ear cress) PE = 1 SV = 1	Putative transcription factor MYB95 [*Arabidopsis thaliana*]	No
	Bo9g164230	K09422|0.0|ath:AT5G15310|ATMYB16 (MYB DOMAIN PROTEIN 16)	Transcription factor MYB76 GN = MBK20.16 OS = *Arabidopsis thaliana* (Mouse-ear cress) PE = 1 SV = 1	Unnamed protein product [*Thellungiella halophila*]	No
MYB34	Bo7g098110	K09422|1e-164|ath:AT5G60890|MYB34	Transcription factor MYB28 GN = MFB13.22 OS = *Arabidopsis thaliana* (Mouse-ear cress) PE = 1 SV = 1	Myb transcription factor *BoMyb34* [*Brassica oleracea* var. *viridis*]	No
	Bo9g014380	K09422|1e-151|ath:AT5G60890|MYB34	Transcription factor MYB28 GN = MFB13.22 OS = *Arabidopsis thaliana* (Mouse-ear cress) PE = 1 SV = 1	MYB domain protein 34-1 [*Brassica rapa* subsp. *pekinensis*]	No
	Bo2g161170	K09422|1e-149|ath:AT5G60890|MYB34	Transcription factor MYB28 GN = MFB13.22 OS = *Arabidopsis thaliana* (Mouse-ear cress) PE = 1 SV = 1	MYB domain protein 34-2 [*Brassica rapa* subsp. *pekinensis*]	No
	Bo2g161180	K09422|1e-150|ath:AT5G60890|MYB34	Transcription factor MYB32 GN = MYB32 OS = *Arabidopsis thaliana* (Mouse-ear cress) PE = 2 SV = 1	MYB34-3 protein [*Brassica rapa* subsp. *pekinensis*]	No
MYB51	Bo5g025570	K09422|1e-163|ath:AT1G18570|MYB51	Transcription factor MYB28 GN = MFB13.22 OS = *Arabidopsis thaliana* (Mouse-ear cress) PE = 1 SV = 1	MYB domain protein 51-2 [*Brassica rapa* subsp. *pekinensis*]	No
	Bo8g067910	K09422|0.0|ath:AT1G18570|MYB51	Transcription factor MYB29 GN = MBK20.15 OS = *Arabidopsis thaliana* (Mouse-ear cress) PE = 1 SV = 1	MYB domain protein 51-2 [*Brassica rapa* subsp. *pekinensis*]	No
MYB122	Bo2g080900	K09422|1e-161|ath:AT1G74080|MYB122	Transcription factor MYB28 GN = MFB13.22 OS = *Arabidopsis thaliana* (Mouse-ear cress) PE = 1 SV = 1	Myb domain protein 122 [*Arabidopsis thaliana*]	No
	Bo6g118350	K09422|1e-164|ath:AT1G74080|MYB122	Transcription factor MYB29 GN = MBK20.15 OS = *Arabidopsis thaliana* (Mouse-ear cress) PE = 1 SV = 1	MYB122 [*Arabidopsis lyrata* subsp. *lyrata*]	No

**Figure 3:**
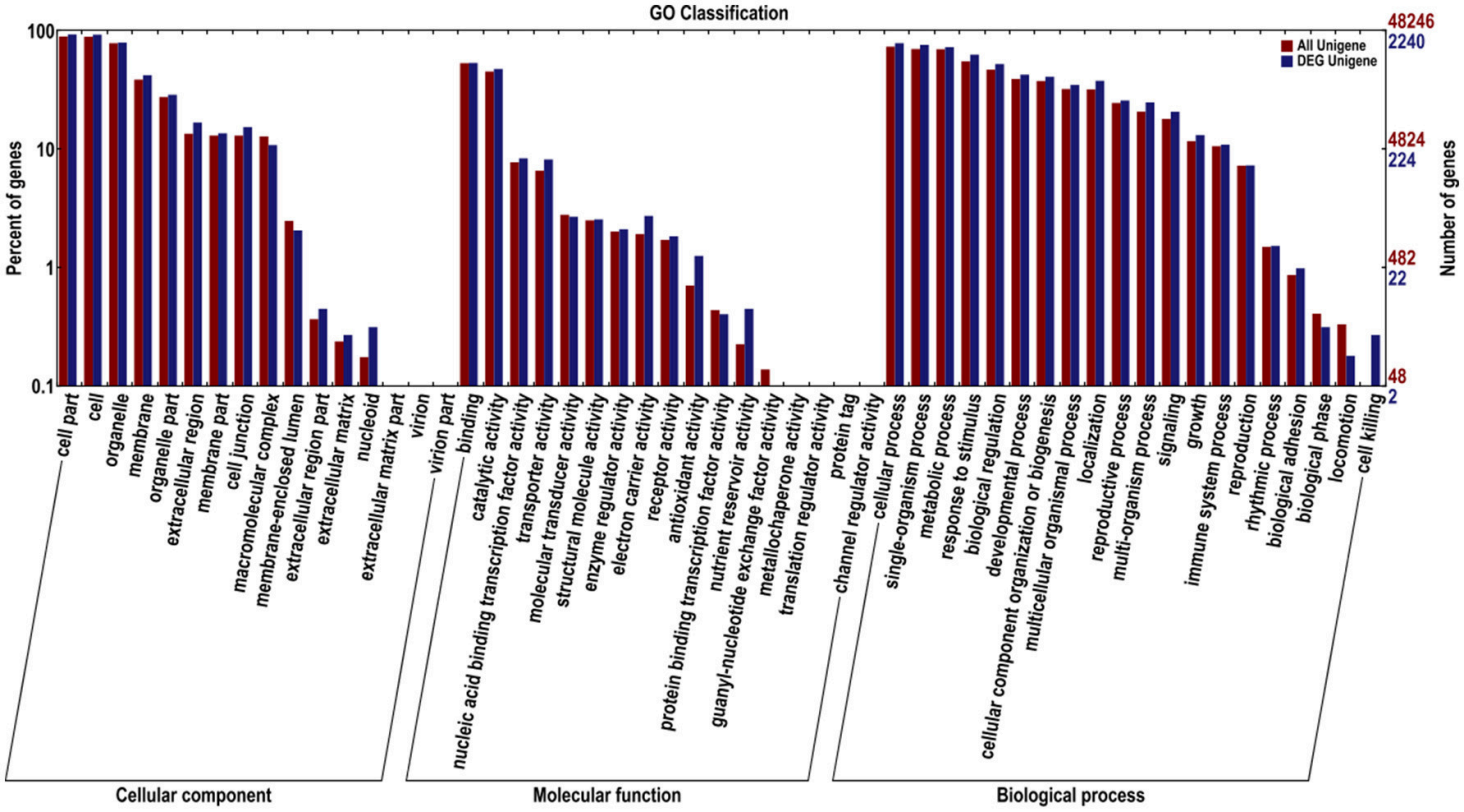
**Gene Ontology (GO) functional classification of all Unigenes and differentially expressed (DEG) Unigenes in JL-08 and JL-09 sprouts**. GO analysis was performed for the three main categories (cellular component, molecular function, and biological process). The *X* axis represented different categories of all Unigenes and differentially expressed Unigenes in JL-08 and JL-09 sprouts. The right *Y* axis indicates the numbers of all Unigenes and differentially expressed Unigenes in a specific category. The left *Y* axis indicates the percentage of a specific category of all Unigenes and differentially expressed Unigenes in a specific category. Red column, all Unigenes; Blue column, DEG Unigenes.

**Figure 4:**
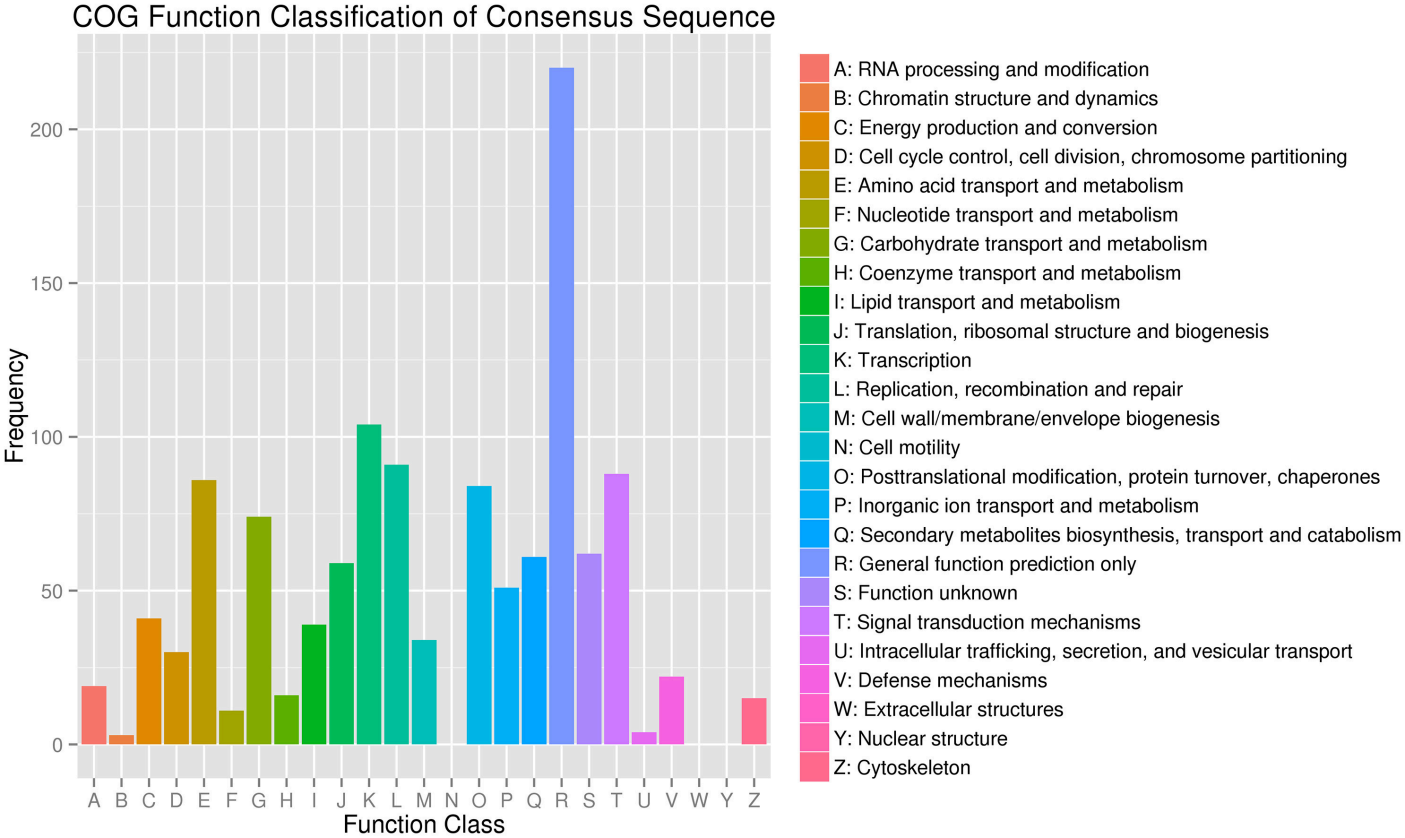
**Clusters of Orthologous Groups (COG) functional classification of differentially expressed genes in JL-08 and JL-09 sprouts**. The *X* axis represented different categories of COG in JL-08 and JL-09 sprouts. The *Y* axis is the frequency of different categories of COG.

We classified these DEGs according to GO and COG to identify their functions (Figures 3, 4). In the GO classification analysis, 2240 genes were assigned to the three GO domains and to 52 terms, with many genes assigned to more than one term. We calculated the percentage of DEGs assigned to each term. The term with the highest percentage in the “cellular component (2124)” domain was “cell part” (DEGs accounting for 97.98% associated with this term), followed by “cell” (97.60%), “organelle” (83.62%), and “membrane” (44.35%). The term with the highest percentage in “biological process (1967)” was “cellular process” (89.32%), followed by “single-organism process” (86.43%), “metabolic process” (82.71%), and “response to stimulus” (71.43%). The term with the highest percentage in “molecular function (1721)” was “binding” (69.49%), followed by “catalytic activity” (61.82%), “nucleic acid binding transcription factor activity” (10.87%), and “transporter activity” (10.63%; Figure 3).

COG functional classification analysis showed that the DEGs were distributed across 22 COG categories. The category with the largest percentage was “transcription,” followed by “replication, recombination, and repair,” “Signal transduction mechanisms,” and “Amino acid transport and metabolism” (Figure 4).

**Figure 5:**
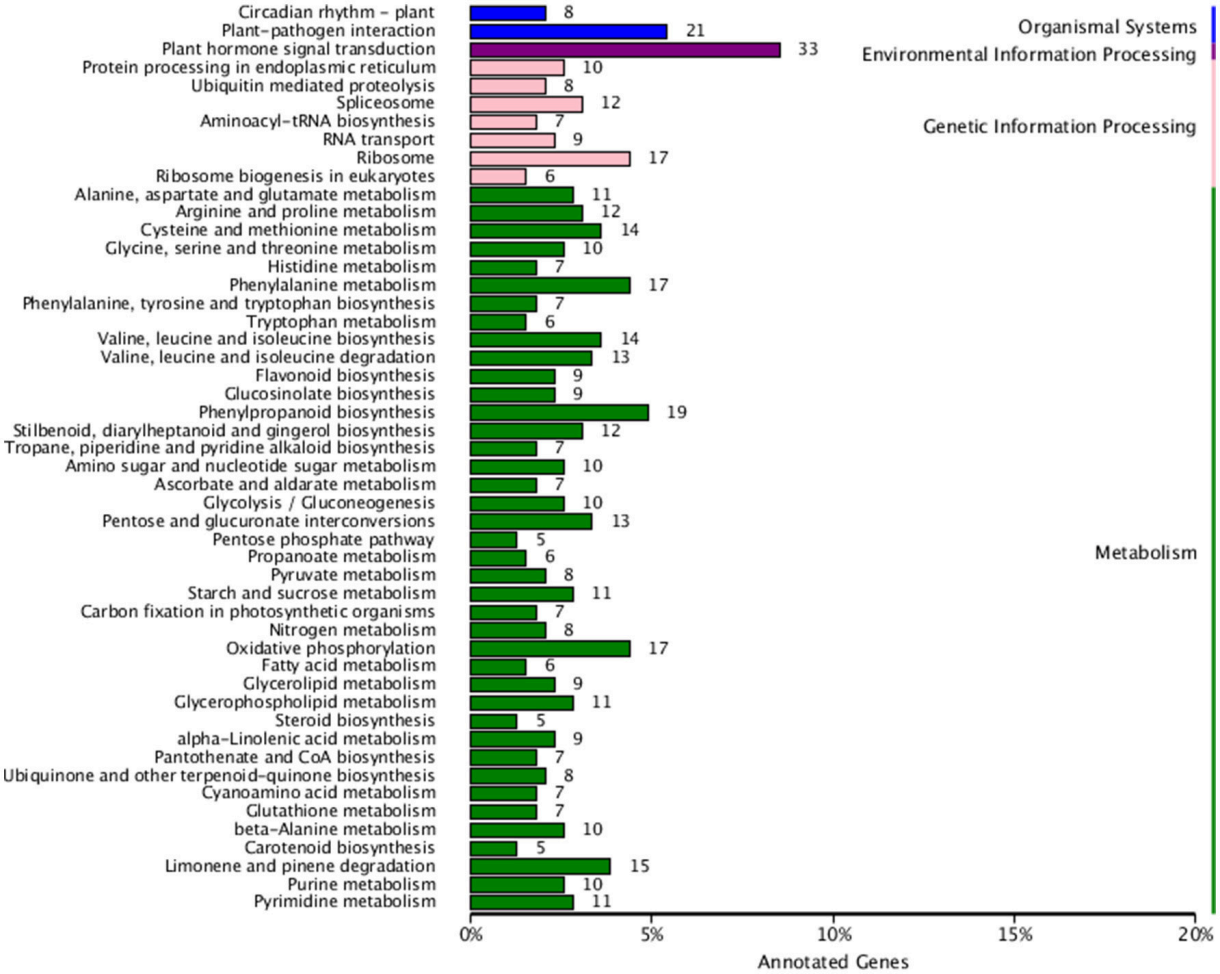
**Kyoto Encyclopedia of Genes and Genomes (KEGG) functional classification of differentially genes expressed in JL-08 and JL-09 sprouts**. The *X* axis is the rate of annotated genes to different categories of KEGG. The *Y* axis represented different categories of KEGG in JL-08 and JL-09 sprouts. Blue column, organismal systems; Purple column, Environmental information processing; Pink column, Genetic information processing; Green column, Metabolism.

**Figure 6:**
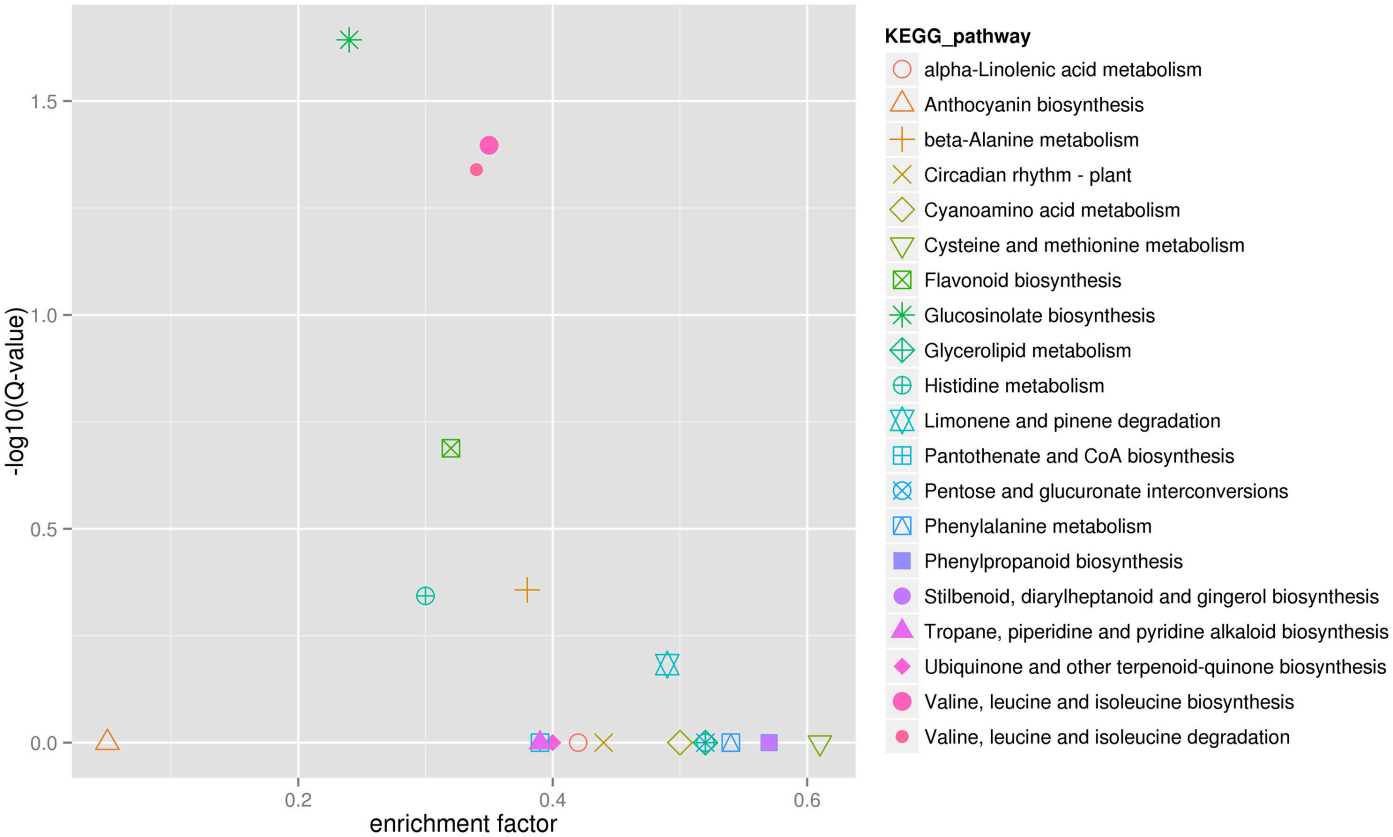
**KEGG enrichment of differentially expressed genes in JL-08 and JL-09 sprouts**. The *X* axis is the enrichment factor of different KEGG pathway. The *Y* axis represented different *Q*-value of different KEGG pathway. The different symbols represented for different KEGG pathway.

Finally, in order to better understand biological pathways in *B. alboglabra*, we used the Kyoto Encyclopedia of Genes and Genomes (KEGG; Kanehisa et al., 2008) database to categorize gene functions with an emphasis on biological pathways. A total of 7563 unigenes were assigned to 105 pathways. The pathways with the largest numbers of unigenes were “plant hormone signal transduction” (ko04075, 509 unigenes, 6.73%), followed by “ribosome” (ko03010, 502, 6.63%), “oxidative phosphorylation” (ko00190, 332, 4.39%), “plant-pathogen interaction” (ko04626, 320, 4.23%), “spliceosome” (ko03040, 319, 4.21%), “protein processing in endoplasmic reticulum” (ko04141, 288, 3.80%), “RNA transport” (ko03013, 280, 3.7%), “purine metabolism” (ko00230, 251, 3.32%), “starch and sucrose metabolism” (ko00500, 236, 3.12%), and “ubiquitin mediated proteolysis” (ko04120, 224, 2.96%). Of these unigenes, 387 were differentially expressed. “Plant hormone signal transduction” contained the most DEGs (33, 8.53%), followed by “plant-pathogen interaction” (21, 5.43%), and “phenylpropanoid biosynthesis” (19, 4.9%; Figure 5). In addition, 42 genes were annotated to the GS biosynthesis pathway (ko00966), with nine DEGs in the Chinese kale varieties with different GS content. The enrichment factor of GS biosynthesis was the smallest and its *Q*-value was the highest of all the KEGG pathways (Figure 6), indicating that these two Chinese kale sprouts do have different GS content.

### Genes related to GS biosynthesis and degradation in chinese kale sprouts

The biosynthesis of GS includes three independent stages: elongation of aliphatic GS chain, formation of core structure, and modification of side chain (Table 3).

**Figure 7:**
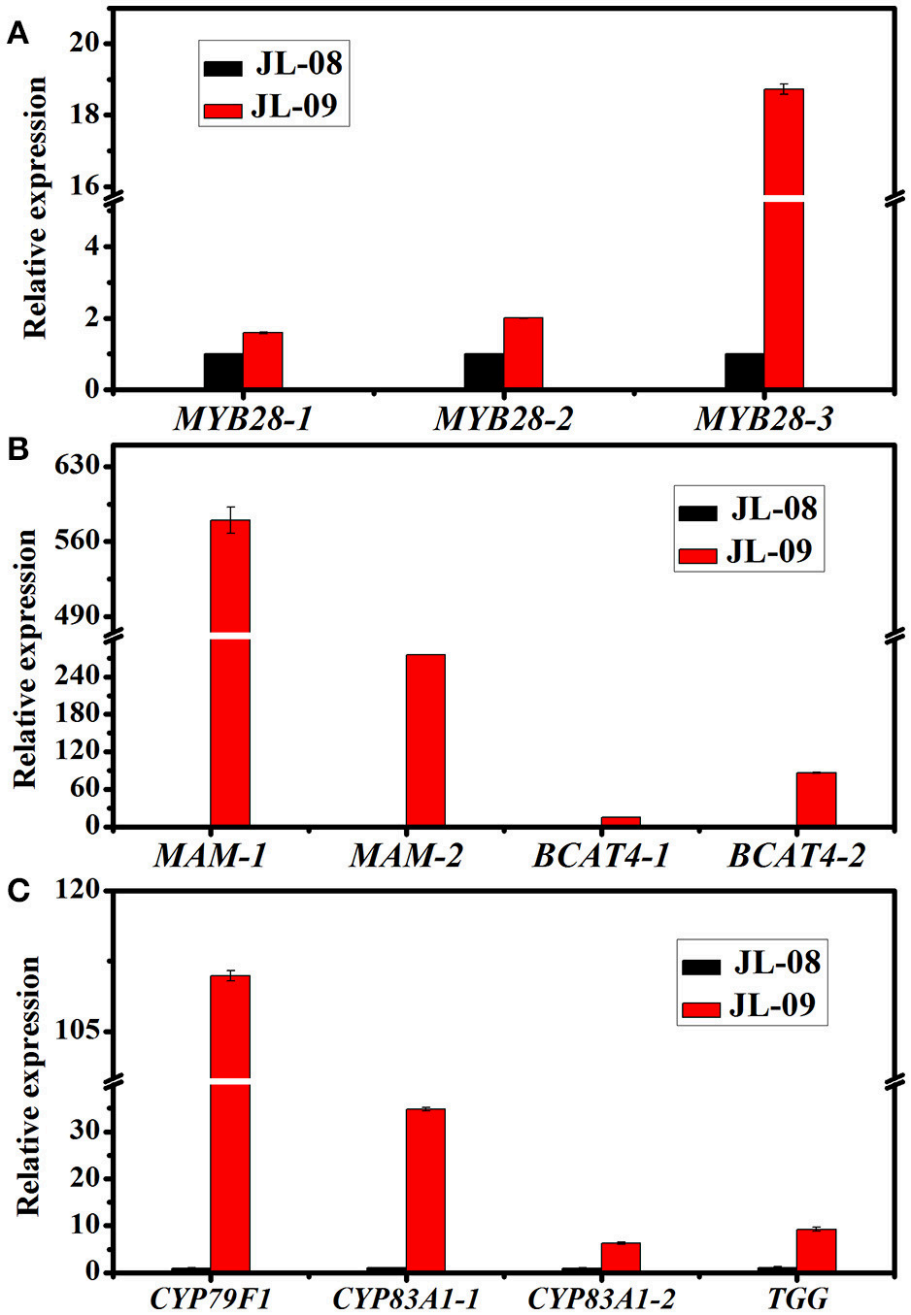
**The expression of 11 differently expressed genes in JL-08 and JL-09 sprouts. (A)** The expression of transcription factors MYB 28-1, MYB28-2, and MYB28-3. **(B)** The expression of side chain elongation genes *MAM1, MAM3, BCAT4-1*, and *BCAT4-2*. **(C)** The expression of core structure biosynthetic genes *CYP79F1, CYP83A1-1*, and *CYP83A1-2*, as well as degradation gene *TGG*. The *X*-axis represented for different glucosinolate biosynthetic genes. The *Y*-axis is the relative expression of one specific gene to reference gene *actin2* and the expression of genes in JL-08 was set to “1” as control. Black column, JL-08; Red column, JL-09.

Three genes related to chain elongation, *branched-chain aminotransferase 4* (*BCAT4*), methylthioalkylmalate synthase (*MAM*), and *branched-chain aminotransferase 3* (*BCAT3*) were identified in Chinese kale sprouts. The expression levels of these genes varied between JL-08 and JL-09. BCAT4, catalyzes the initial step of methionine chain elongation by deaminating methionine to 4-methylthio-2-oxobutanoate. In JL-08, the expression of *BCAT4* was low, while two orthologs of *BCAT4*, Bo3g073430, and Bo5g113720, were detected in JL-09. A similar expression pattern was found for the gene *MAM*, with higher expression levels in JL-09 compared with JL-08. *MAM* (IPMS-like genes) are involved in the condensation of deaminated methionine with acetyl-CoA. BCAT3 catalyzes the last step in the process of chain elongation. *BCAT3* was detected both in JL-08 and JL-09 with no significant difference in its expression level. We validated the result of sequencing data using qPCR of *BCAT4, MAM*, and *BCAT3*, and the results show a similar expression pattern, with JL-09 showing higher expression levels of *BCAT4* and *MAM* and comparable counts of *BCAT3* compared with those in JL-08 (Figure 7B).

We identified nine genes functioning in GS core structure formation in Chinese kale sprouts (Table 3). The committed step in formation of the basic glucosinolate skeleton is conversion of (n)homomethionine, tryptophan, or phenylalanine into the corresponding oximes by the cytochrome P450 mono-oxygenases CYP79F1, CYP79B2, and CYP79B3, or CYP79A2, respectively (Chen et al., 2003). The aldoximes are then converted to S-alkylthiohydroximate intermediates by CYP83A1 and CYP83B1, and cleaved by a C-S lyase SUR1 into thiohydroximates, followed by sequential glucose (UGT74C1) and sulfate transfer (SOT) to complete the basic glucosinolate skeleton. In our experiment, no expression of *CYP79F1* was detected in high-GS JL-08, while in low-GS JL-09 the expression value was 30.77 FPKM. The count of another GS core structure formation gene, *CYP83A1*, was also much higher in JL-09 compared with JL-08 (Figure 7C).

The alkenyl/hydroxypropyl (AOP) locus plays important roles in the modification of side chains. There are three alleles of AOP, controlling the modification of side chain (Kliebenstein et al., 2001; Yan and Chen, 2007). Only the *Alk* and *OHP* loci, which are responsible for converting methylsulfinylalkyl GSs into alkenyl and hydroxyalkyl GSs, respectively, had significant control over accumulation of GS in *Arabidopsis* (Kliebenstein et al., 2001). In the Col-0 line, AOP comprises of two tandem genes, *AOP2* and *AOP3*. However, no AOP expression was detected in Chinese kale sprouts.

In *Arabidopsis, PEN2, TGG1*, and *TGG2* are three myrosinases genes involved in degradation of GS. Among these, PEN2 functions to cleave indolic GSs. TGG1 and TGG2 have long been known as important myrosinases (Gao et al., 2014). Aliphatic GSs cannot be degraded in *tgg1tgg2* double mutant while indolic GSs can be reduced slightly, indicating that TGG1 and TGG2 mainly degrade aliphatic GSs and have only slight effects on indolic ones. In the sequenced tissues, five orthologs of myrosinase genes (Bo8g039420, Bo00934s010, Bo2g155810, Bo9g022660, and Bo14804s010) were identified based on the NCBI database (Table 3). Only the expression of Bo8g039420 was decreased in JL-09 compared with that in JL-08.

### Transcription factors related to GS biosynthesis in chinese kale sprouts

In *Arabidopsis, MYB28, MYB29*, and *MYB76 are commonly defined as* transcriptional regulators in the biosynthesis of aliphatic GS, which can specifically activate aliphatic GS biosynthesis related genes i.e., *MAM3, CYP79F1*, and *CYP83A1* (Gigolashvili et al., 2007b, 2008). In contrast, *MYB34, MYB51*, and *MYB122* exclusively trans-activate the promoters of *TSB1, CYP79B2*, and *CYP79B3*, which are involved in the indolic GS biosynthetic pathway (Gigolashvili et al., 2007a). We identified transcription factors regulating the biosynthesis of GS, homologous to *MYB28, MYB29, MYB76, MYB34, MYB51*, and *MYB122*, in Chinese kale sprouts (Table 4). After annotation using NCBI and Swissprot, 14 genes were classed as transcription factors related to GS biosynthesis in Chinese kale. Among these, expression counts of three MYB28 homologs were higher in JL-09 compared with those in JL-08, while other MYBs showed no significant differences between the two varieties. Analysis using qPCR also showed that the expression levels of transcription factor *BaMYB28* were higher in JL-09 than in JL-08, consistent with the sequencing data (Figure 7A).

## Discussion

### 4C GSs predominant in *Brassica alboglabra* sprouts

The high GS content of in *Brassica* sprouts compared with mature tissues, especially their much higher content of aliphatic GS, has attracted attention in the past decade (Fahey et al., 1997; Guo et al., 2011; Wei et al., 2011; Qian et al., 2016). The molecular basis of this trait, and especially whether GS metabolic genes are involved in this process, remains unknown. In our experiment, 37 genes were annotated as putatively related to GS biosynthesis, degradation, and regulation, of which 14 were identified as transcription factors, 18 as biosynthetic genes and five encoded catabolic enzymes. *Brassica* sprouts contain mainly aliphatic GSs (>95% of the total GS). Based on side chain length, the aliphatic GSs can be classified into 3C (propyl), 4C (butyl), and 5C (pentyl) GSs, increasing the complexity of GS biosynthesis. Brassica species contain various combinations of the above three types of aliphatic GS (Gland et al., 1981; Ishida et al., 2014). Sinigrin (2-propenyl) and GIB (3-methylsulfinylpropyl), which are the main GSs in cauliflower, are 3C GSs. The 4C GSs GNA (3-butenyl), PRO (2-hydroxy-3-butenyl), and glucoraphanin (GRA, 4-methylsulfinylpropyl) are predominant in Chinese kale, rapeseed, and broccoli, respectively. Glucobrassicanapin contains five carbons in its side chain and is mainly found in *B. rapa*. Mostly 3C and 4C GS are detected in *B. oleracea* crops (Sharma et al., 2016). In our study, the most abundant GSs are 4C GSs including GNA and PRO.

The chain-elongation pathway in aliphatic GS biosynthesis is believed to have evolved from leucine biosynthesis, due to similarities in phylogeny and catalytic abilities of MAM and isopropylmalate synthase (IPMS), an enzyme involved in leucine biosynthesis, as well as the function of BCATs (De Kraker and Gershenzon, 2011). Polymorphisms at MAM alleles in *Brassicaceae* may be responsible for their diversity of aliphatic GS concentration and profile (Field et al., 2006), and *MAM* in *Brassica* does not belong to any of the *MAM* subclasses found in *Arabidopsis* and its close relatives (Benderoth et al., 2009). Aliphatic GSs derived from both homomethionine (3C) and dihomomethionine (4C) can be accumulated in *Brassica* accessions, while in *A. thaliana* aliphatic GSs can only be generated either from homomethionine or dihomomethionine but not both (Velasco and Becker, 2000; Benderoth et al., 2009). In Chinese kale sprouts, GIB (3C), PRO (4C), and GNA (4C) were all detected, indicating the co-existence of homomethionine and dihomomethionine. This is similar to other members of the *Brassica* genus, in which aliphatic GSs can be generated from homo-, dihomo-, and trihomo-methionine (Velasco and Becker, 2000). Two members of MAM1 (Bo2g161100 and Bo7g098000) in Chinese kale sprouts aligned to genes in *B. oleracea* and *B. juncea*, respectively. Three MAM genes (*MAM1* [*At5g2310*], *MAM2*, and *MAM3* [*At5g23020*]) have been detected in different *Arabidopsis* ecotypes (Benderoth et al., 2009). Variation in MAM is one cause of biochemical polymorphism of GS in Chinese kale and *Arabidopsis*.

Another gene believed to be involved in the formation of GS diversity in *Brassica* is *BoGSL-PRO*, which is reported to be involved in the production of homomethionine-derived GSs (Gao et al., 2006). Although IPMS plays a key role in the process of leucine biosynthesis, metabolite analyses of *BatIMS* (*IPMS* gene from *Brassica*) overexpression mutant show that amino acid metabolism are changed and GSs content are increased in the overexpressed plants (Field et al., 2006). In Chinese kale spouts, two genes, Bo2g161100 and Bo7g098000, aligned to *IPMS* of *Brassica* specie*s* by NCBI and were also classified into the KEGG ortholog K01649 along with *Arabidopsis MAM* (Table 3), indicating the similarity of these two genes. It has been reported that *MAM* and *IPMS* have similar gene structures and mostly identical intron positions in *Arabidopsis* (Kroymann et al., 2001). Two functional *IPMSs* as well as three *MAMs*, sharing 60% amino acid identity, have been detected in different *Arabidopsis* ecotypes (Benderoth et al., 2009), which both catalyze the condensation of acetyl-CoA and 2-oxo acids but with different substrate scopes. MAM uses 2-oxo acid and its derivatives whereas IPMSs only use 2-oxoisovalerate (De Kraker and Gershenzon, 2011).

BCATs catalyze the final transamination reactions that convert 2-oxo acids into branched-chain amino acids. Among the seven identified BCATs in *Arabidopsis*, BCAT4 and BCAT3 have been shown to participate in the initial and terminal steps, respectively, of side-chain elongation in the biosynthesis of methionine aliphatic GS (Angelovici et al., 2013). In Chinese kale sprouts, BCAT4 (Bo3g073430 and Bo5g113720) and BCAT3 (Bo1g080200 and Bo8g078930) were annotated to *B. rapa* and *A. lyrata*.

Other than the side chain elongation genes, one gene *CYP79F2* participating in formation of GS core structure in *Arabidopsis* is closely related to biosynthesis of long-chain aliphatic GSs. The knockout mutant *cyp79f2* (knockout of *CYP79F2*) shows significantly reduced long-chain aliphatic GS content (Chen et al., 2003). In Chinese kale, *CYP79F2* is absent, which corresponds well with the fact that 3C and 4C GSs are the predominant aliphatic GSs in Chinese kale. *CYP79F2* was also found to be absent in *B. rapa* by comparative analyses of *A. thaliana* and *B. rapa* on a genome-wide level (Wang et al., 2011), indicating differences in the GS biosynthetic pathway in *A. thaliana* and *Brassica* crops.

### High expression level of GS biosynthetic genes with low GS content in JL-08 sprouts

**Figure 8:**
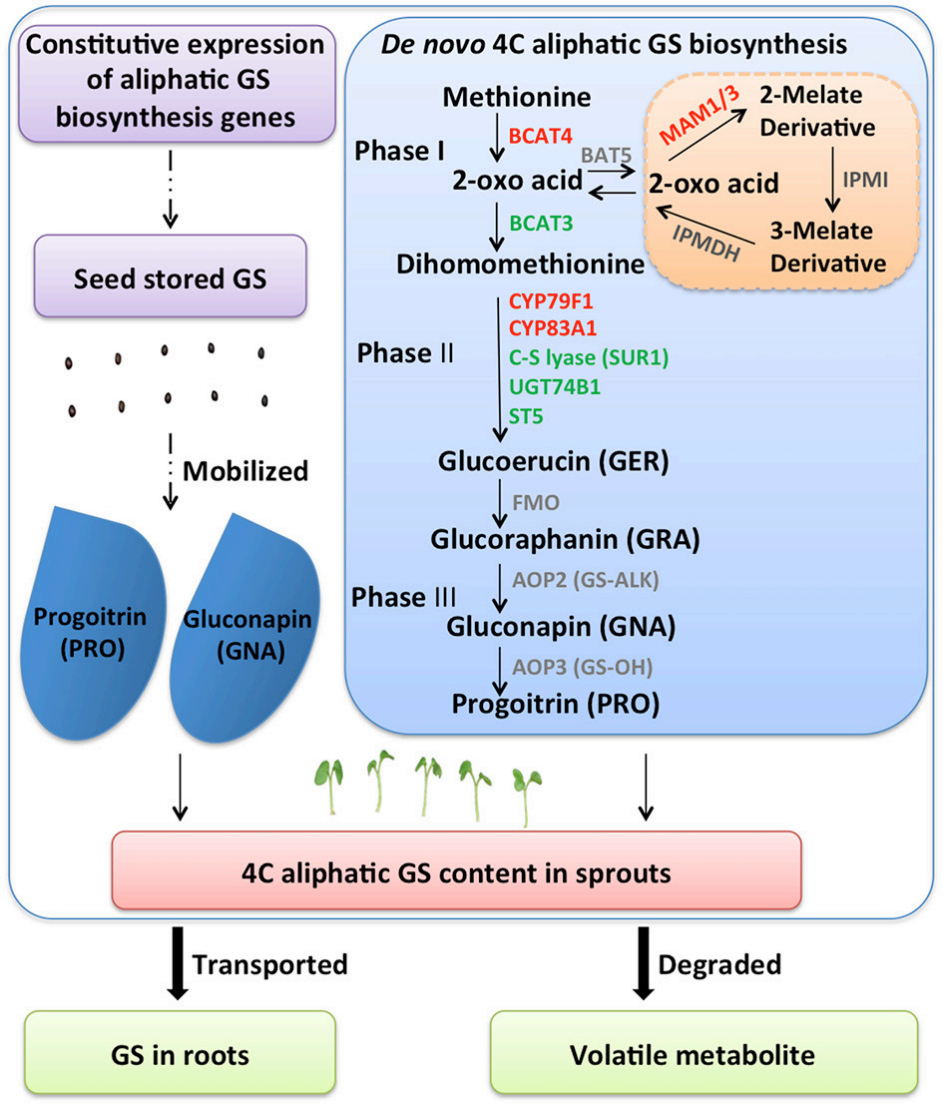
**A diagram showing possible GS metabolisms mechanism in *B. alboglabra* sprouts**. GS contents in sprouts are determined by dynamic equilibrium of seed stored GS mobilization, *de novo* synthesis, degradation, and extra transport. Phase I: side chain elongation of GS biosynthesis; Phase II: core structure formation of GS biosynthesis; Phase III: side chain modification of GS biosynthesis. The solid arrow represents for GS biosynthetic pathway, the dash arrow for unknown pathway, the bold arrow for transport and breakdown of GS. GS, glucosinolate; GER, glucoerucin; GRA, glucoraphanin; GNA, gluconapin; PRO, progoitrin.

Selecting optimal sprouting condition or supplementing with chemicals is used to enhance GS content in Brassica sprouts. In terms of GS metabolic regulation, increasing expression levels of biosynthetic genes is a conventional approach. However, in sprouts, other conditions should be considered. In our research, with two Chinese kale varieties differing in GS content, lower expression levels of GS biosynthesis genes (*BCAT4, MAM, CYP79F1, CYP79B3*, and *CYP83A1*) were found in JL-08 (high-GS variety) compared with JL-09 (low-GS variety). This is astonishing because it is generally believed that high-GS JL-08 must have higher gene expression levels. Further analysis of GS accumulation in sprouts has been done, and four processes are responsible for maintaining GS content in sprouts: (i) *de novo* biosynthesis of GS in sprouts; (ii) GS released from seeds; (iii) degradation of GS; and (iv) transported to root (Figure 8).

By analyzing RNA-seq data of JL-08 and JL-09 sprouts, we have found that many key genes for GS formation expressed with very low level, for example *bile acid transporter* (*BAT5*) and *AOP2*. The *BAT5* is responsible for the transportation of short- and long-chain 2-keto acids, which are intermediate products in the process of methionine-derived GS biosynthesis. *BAT5* expressed highly in seedlings and mature plants (Gigolashvili et al., 2009). AOP2 is directly responsible for biosynthesis of the alkenyl GS GNA. Three orthologs of *AOP* loci have been found in the genome of *B. rapa*, which corresponds well with the fact that the dominant GS in *B. rapa* are alkenyl GSs s (Wang et al., 2011). AOP2 (or GS-ALK) catalyzes the conversion of desirable GRA to deleterious GNA and PRO, which are present in very high amounts in most of the cultivable Brassica species. Transformation with the antisense *AOP2* in Chinese kale and constitutive silencing of *GSL-ALK* homologs in *B. juncea* have been attempted with the aim of changing the proportions of GNA and GRA (Augustine and Bisht, 2015; Qian et al., 2015). However, we detected no transcripts of *BAT5* and *AOP2* in Chinese kale sprouts, indicating either that there is no intact GS biosynthesis pathway in sprouts, or that biosynthesis is initiated but the transportation of GS (*BAT5*) and modification of side chains (*AOP2*) has not been activated. Thus, regulation of GS in sprouts should not focus on increasing the expression levels of biosynthesis genes.

Another reason lies in the source of GS in sprouts. During the first seedling establishment stage, the seedling is dependent on the seed's energy reserves. When seeds are formed, most plants store a food reserve within the seed, containing starch, proteins, and oils. This food reserve provides nourishment to the growing embryo. When the seed imbibes water, hydrolytic enzymes are activated that break down these stored food resources into metabolically useful chemicals. GS is also stored in the seed of Chinese kale, and during germination, it is hydrolyzed to supply sulfur for primary metabolism. Thus, GS content in sprouts may exhibit a decreasing tendency because of its sulfur-donor role during germination. In our experiment, the different GS content in JL-08 and JL-09 sprouts is mostly due to varying GS content in their seeds (Supplementary Figure 1). Comparison of the GS profiles of mature seeds with those of cotyledons indicates that GS in seed mainly stored in the cotyledons and transferred continuously during growth (Petersen et al., 2002).

A third reason may lie in the high expression levels of GS metabolic gene TGG in JL-09, which can catalyze the degradation of GS. The RNA-seq data combined with qPCR result show that the expression of *TGG* (Bo8g039420) in JL-09 was significantly up-regulated compared with that in JL-08. It has also been reported that expression of *TGG2* in broccoli sprouts expressed is astonishingly higher (20–130 times) after germination, and high expression of GS metabolic genes is accompanied by decreased concentration of GS (Gao et al., 2014). In addition, the expression of myrosinase-binding protein (MBP) is highest in germinating seedlings of *B. napus*. GSs and MBP are probably co-localized in the *B. napus* seed. However, the content of aliphatic GSs decreased dramatically during germination with no MBP detected outside myrosin cells (Andréasson et al., 2001). The degradation of GSs by myrosinase releases glucose and sulfate, suggesting that these are of nutritional value for developing seedlings (James and Rossiter, 1991).

The last possible way to change GS content in sprouts is transport from sprouts to roots. As the distinct GS profiles existed in plant sprouts and roots, i.e., aliphatic GS mainly in sprouts and indolic GS mainly in roots, and no transformation were found between aliphatic and indolic GS, the transport of GS in sprouts stage may have no effect in GS content in sprouts.

In conclusion, the GS accumulated in sprouts may come from seed storage and/or be synthesized *de novo*. As low expression levels of GS metabolic genes were found in JL-08 sprouts that accumulated a higher content of GS, we speculate that lower levels of catabolic enzyme myrosinase *TGG* and higher levels of seed GS in JL-08 result in higher GS content in its sprouts. Thus, the endeavor to enhance the nutritional quality of sprouts with regard to GS content may focus on reducing the activity of the catabolic enzyme myrosinase or breeding for high-GS-content varieties for sprout production.

## Author contributions

ZL and XX conceived of the study and helped to revise the manuscript. RG conceived of the study, conducted the experiment(s) and wrote the manuscript. ZH, YD, and XC prepared materials and conducted qRT-PCR analysis. All authors read and approved the final version of the manuscript.

### Conflict of interest statement

The authors declare that the research was conducted in the absence of any commercial or financial relationships that could be construed as a potential conflict of interest.
