# Supplementary material for: Comparative Transcriptome Analyses Reveal a Special Glucosinolate Metabolism Mechanism in Brassica alboglabra Sprouts
Source: Front Plant Sci. 2016 Oct 4;7:1497. doi: 10.3389/fpls.2016.01497 (PMC5047911; doi:10.3389/fpls.2016.01497)
Supplement: Supplementary file 1 [file Table1.DOCX]

| **Comparative transcriptome analyses reveal glucosinolate metabolism mechanism in *Brassica alboglabra* sprouts Rongfang Guo, Zhongkai Huang, Yanping Deng, Xiaodong Chen, Xu XuHan*, Zhongxiong Lai*** | | | |
| --- | --- | --- | --- |
| **Supplementary Table S1 Primers used for qPCR** | | | |
| Gene name | primers |  |  |
| MYB28-1F | GCCAAGGAGATGGATAATCG |  |  |
| MYB28-1R | TGTTCAGGAGACAAGCCGA |  |  |
| MYB28-2F | TCGGACATAGCGAGACATTTAC |  |  |
| MYB28-2R | TAAGACGAGACAGGGAAGGC |  |  |
| MYB28-3F | CTCATAAACACTCTCGCCGA |  |  |
| MYB28-3R | ACTTCTTGGGACACATAGGACA |  |  |
| MAM-1F | TTACTCCACCGCAGAAGATAGA |  |  |
| MAM-1R | ACTCCCAAGCCGCCTTTAT |  |  |
| MAM-2F | GCTACCTCAAAGCAAACACTCC |  |  |
| MAM-2R | TCCTCGGCATTTCAAAGC |  |  |
| BCAT2-1F | ATAGCCACAGCATTGCGA |  |  |
| BCAT2-1R | AACATCTTTCTCCACAGAGCAG |  |  |
| BCAT2-2F | TCAAGGTGAACTCAGTCGCT |  |  |
| BCAT2-2R | CAACCGAAGGAGAAGGCAT |  |  |
| BCAT4-1F | TAGCAGAGGCGAAAGCACA |  |  |
| BCAT4-1R | CCTTGTAGCCGAAATCACG |  |  |
| BCAT4-2F | TGGATAACATTAGCAGAGGCG |  |  |
| BCAT4-2R | CAACCTTGTAGCCGAAATCAC |  |  |
| CYP79F1-F | ATTGAAGCGGACAACCTCC |  |  |
| CYP79F1-R | GAGAGAAACTCGGCAAACAGT |  |  |
| CYP83A1-1F | GCAATGAACCACTACACACCG |  |  |
| CYP83A1-1R | ACTTCTTCCCGAACGCTTG |  |  |
| CYP83A1-2F | ATCGCTGGCTACGACATACC |  |  |
| CYP83A1-2R | GCTGAACCAAGACGCATTC |  |  |
| TGG-F | CACTGGGACCTTCCTCAAGT |  |  |
| TGG-R | CAACCATTGGAGAACATCTACC |  |  |
