# Supplementary material for: Comparative Transcriptome Analyses Reveal a Special Glucosinolate Metabolism Mechanism in Brassica alboglabra Sprouts
Source: Front Plant Sci. 2016 Oct 4;7:1497. doi: 10.3389/fpls.2016.01497 (PMC5047911; doi:10.3389/fpls.2016.01497)
Supplement: Supplementary file 2 [file DataSheet1.DOCX]

**Comparative transcriptome analyses reveal a special glucosinolate metabolism mechanism in *Brassica alboglabra* sprouts**

Rongfang Guo ^a, b^, Xu XuHan ^a, c,*^, Zhongxiong Lai ^a,b,*^

Correspondence:

Prof. Zhongxiong Lai

College of Horticulture

Fujian Agriculture and Forestry University

Fuzhou 350002, China

laizx01@163.com

Prof. Xu XuHan

Institut de la Recherche Interdisciplinaire de Toulouse

Toulouse, France

[xxuhan@163.com](mailto:xxuhan@163.com)

Supplementary Figure 1 The content of glucosinolates in seeds of JL-08 and JL-09. The X-axis represented the profiles of glucosinolate in JL-08 and JL-09 sprouts. GIB: Glucoiberin, PRO: Progoitrin; GNA: Gluconapin; GBS: Glucobrassicin; 4OMGBS: 4-Methoxy glucobrassicin; TGS: Total glucosinolate. The Y-axis is the content of individual and total glucosinolate in JL-08 and JL-09 sprouts. Black column: JL-08; Red column: JL-09. The error bar represents the ± SE.
